# Prevalence of Syphilis among Pregnant Women in Sub-Saharan Africa: A Systematic Review and Meta-Analysis

**DOI:** 10.1155/2019/4562385

**Published:** 2019-07-16

**Authors:** Siraj Hussen, Birkneh Tilahun Tadesse

**Affiliations:** ^1^School of Laboratory Science, College of Medicine and Health Sciences, Hawassa University, Hawassa, Ethiopia; ^2^Department of Pediatrics and Child Health, College of Medicine and Health Sciences, Hawassa University, Hawassa, Ethiopia

## Abstract

**Objective:**

Syphilis is one of the most imperative STIs, caused by the spirochete* Treponema pallidum*. During pregnancy it is associated with disastrous health outcomes in the newborn. In sub-Saharan Africa, study findings on the prevalence of syphilis among pregnant women are highly dispersed and inconsistent. The aim of the current review is to conduct a systematic review and meta-analysis of syphilis in sub-Saharan Africa among pregnant women.

**Design:**

Systematic review and meta-analysis.

**Data Sources:**

Databases including MEDLINE, PubMed, Cochrane Library, Google Scholar, and HINARI and reference lists of previous prevalence studies were systematically searched for relevant literature from January 1999 to November 2018. Results were presented in forest plot, tables, and figures. Random-effects model was used for the meta-analysis. For the purpose of this review, a case of syphilis was defined as positive treponemal or nontreponemal tests among pregnant women.

**Data Extraction:**

Our search gave a total of 262 citations from all searched databases. Of these, 44 studies fulfilling the inclusion criteria and comprising 175,546 subjects were finally included.

**Results:**

The pooled prevalence of syphilis among pregnant women in sub-Saharan Africa was 2.9% (95%CI: 2.4%-3.4%). East and Southern African regions had a higher syphilis prevalence among pregnant women (3.2%, 95% CI: 2.3%-4.2% and 3.6%, 95%CI: 2.0%-5.1%, respectively) than the sub-Saharan African pooled prevalence. The prevalence of syphilis among pregnant women in most parts of the region seemed to have decreased over the past 20 years except for the East African region. However, prevalence did not significantly differ by region and time period.

**Conclusion:**

This review showed a high prevalence of syphilis in sub-Saharan Africa among pregnant women. The evidence suggests strengthening the screening program during pregnancy as part of the care package during antenatal care visits. Programs focusing on primary prevention of syphilis in women should also be strengthened.

## 1. Introduction

Syphilis is one of the most imperative STIs, caused by the spirochete* Treponema pallidum* and it is a significant public health issue, especially in developing countries including sub-Saharan Africa [1]. More than 10 million people are infected with syphilis worldwide; the majority of these infections occur in sub-Saharan Africa and Asia [2]. Annually, around 2 million pregnant women are estimated to have active syphilis infection while only less than one-tenth would be diagnosed and receive the treatment. More than 90% of these infections occur in resource limited settings [3–5]. 

The prevalence of syphilis infection among pregnant women in sub-Saharan Africa is estimated to be 2.7%, which represents nearly 1 million pregnancies to be at risk annually [6]. In the United States, recent data show that more than 30,000 cases of primary and secondary syphilis infection were reported [7]. Unfavorable pregnancy outcomes were reported to be more than four times higher in untreated syphilis infection among pregnant women as compared to pregnant women without syphilis infection [8].

Syphilis is transmitted via sexual contact or from mother to child during pregnancy or at delivery [9]. During pregnancy, syphilis infection might also increase the risk of mother-to-child transmission of HIV in cases where mothers are coinfected [10]. Syphilis in pregnancy which is not treated properly was reported to cause poor pregnancy outcomes in about half of the cases—stillbirths in 40%, deaths and neonatal morbidity in 20%, and small birth weight deliveries in 20% [11–13].

In 2016, the WHO strategy on global health sector outlines the control and prevention of sexually transmitted infections (STI). The goal of the global health sector strategy is to reduce the incidence of syphilis by 90% and congenital syphilis to <50 cases per 100,000 live births in the coming decade [14]. To support implementation and assess progress towards the target of these strategies, understanding the pooled estimates of prevalence of these infections by region and subregion would help to guide clinical decision making, public resource allocation, and optimization of intervention protocols. Currently, different studies done in the region reveal a considerable variation in the prevalence of syphilis among pregnant women including geography and time. Overall, there is lack of a recently summarized data on syphilis among pregnant women in the region. The current review and meta-analysis aims to provide updated information on syphilis infection during pregnancy and pooled estimates of the prevalence of syphilis among pregnant women. 

## 2. Methods

### 2.1. Search Strategy

A systematic literature search was conducted on the prevalence syphilis in sub-Saharan Africa. Relevant citations were identified through a literature search of MEDLINE, PubMed, HINARI, Google Scholar, and Cochrane Library. The search was based on the combination of the following special index search terms using medical subject headings (MeSH) and Boolean operations: “sexually transmitted infections” OR “sexually transmitted diseases” OR “genital tract infections” OR “reproductive tract infections” OR “syphilis” OR “*Treponema palladium*” and “pregnancy” OR “pregnant women” OR “antenatal” OR “prenatal care” and “developing country” OR “Africa” “Lists of particular countries in Sub-Saharan Africa” and “prevalence” OR “epidemiology” from January 1^st^ 1999 to November 20^th^, 2018. Literature search was limited to published studies among humans in English language. All records were managed in Endnote version of X7 (Clarivate Analytics, Philadelphia, PA, USA) and duplicated studies were carefully removed. The search was carried out from April 5^th^, 2018 to November 20, 2018. The Preferred Reporting Items for Systematic Reviews and Meta-Analyses (PRISMA) guideline [15] was used to report the result of this systematic review and meta-analyses (Table S1).

### 2.2. Selection Criteria

Abstracts retrieved from the initial search were screened using defined inclusion and exclusion criteria.

### 2.3. Inclusion Criteria and Exclusion Criteria

Studies were selected for systematic review and meta-analysis if (1) they were conducted in sub-Saharan Africa, (2) study design was cross-sectional, (3) studies reported the prevalence of syphilis, and (4) studies reported data in humans and were published in the English language.

Studies were examined for eligibility by reading their titles and abstracts. Relevant abstracts were further assessed for inclusion in the list of full text articles. During the article selection process, studies which did not have full texts were excluded since it was not possible to assess the quality of each article in the absence of their full texts.

#### 2.3.1. Case Definition of Syphilis

In the current review, a case of syphilis was considered when one or more of TPHA (Treponema Hemagglutination), EIA (Enzyme Immunoassay), VDRL (Venereal Disease Research Laboratory), RPR (Rapid Plasma Reagin), ELISA (Enzyme Linked Immunosorbent Assay), or RICT (Rapid Immunochromatographic test) were positive among pregnant women. All articles which used treponemal (TPHA, EIA), nontreponemal (VDRL and RPR), or other tests to make a diagnosis of syphilis were included in the review.

### 2.4. Data Extraction

Two researchers (SH and BTT) independently extracted the data from included studies using a standardized and pretested format prepared in Microsoft Excel. The data abstraction format included first author, study design, region in sub-Saharan Africa, publication year, sample size, study population, number of individuals who tested positive, and prevalence of syphilis. Disagreement on data extractions between researchers was resolved through discussion and consensus.

### 2.5. Quality Assessment

The quality of each article was assessed using 9 point Joanna Briggs Institute (JBI) critical appraisal tools. The tool uses the following criteria.

(1) Was the sample frame appropriate to address the target population? (2) Were study participants sampled in an appropriate way—was sampling method appropriate to the design? (3) Was the sample size adequate—have the authors used appropriate assumptions to calculate sample size? (4) Were the study subjects and the setting described in detail? (5) Was the data analysis conducted with sufficient coverage of the identified sample as coverage bias can occur when not all subgroups of the identified sample respond at the same rate? (6) Were valid methods used for the identification of the condition? (7) Was the condition measured in a standard, reliable way for all participants? (8) Was there appropriate statistical analysis? (9) Was the response rate adequate?

Individual studies were assigned a score that was computed using different parameters in line with the review objectives. The responses were scored 0 for “Not appropriate and not reported” and 1 for “Yes”. Total scores ranged between 0 and 9. Studies with medium (fulfilling 50% of quality assessment parameter) and high quality were included for analysis [16]. All the studies scored at least 50% and none of them were excluded based on the quality assessment criteria (Table S2).

### 2.6. Statistical Analysis

The data analyses were done using STATA (version.14.0) software. Forest plot, figures, and tables were used to describe the included original articles. A random effect model was used to compute the pooled prevalence of syphilis as there was heterogeneity among studies. The estimated pooled prevalence was presented with 95% confidence interval (CI).

### 2.7. Subgroup Analysis

Subgroup analysis was performed based on region (East, West, Central,and Southern Africa) and year of study (1999-2003, 2004-2008, 2009-2013, and 2014-2018).

### 2.8. Heterogeneity and Publication Bias

Cochran's Q test and *I*^2^ statistic were used to assess statistical heterogeneity. *I*^2^ values 25%, 50%, and 75% are classified as low, medium, and high heterogeneity, respectively [17, 18]. We used random-effects meta-analysis to estimate the pooled prevalence with 95% CI as the *I*^2^ >25%. Cochran's Q test–p < 0.10 was used to verify the existence of statistical heterogeneity. To assess publication bias, Begg intercept statistics test was used; a p value < 0.05 suggests presence of a statistically significant publication bias [19]. 

## 3. Results

### 3.1. Identified Studies

**Figure 1:**
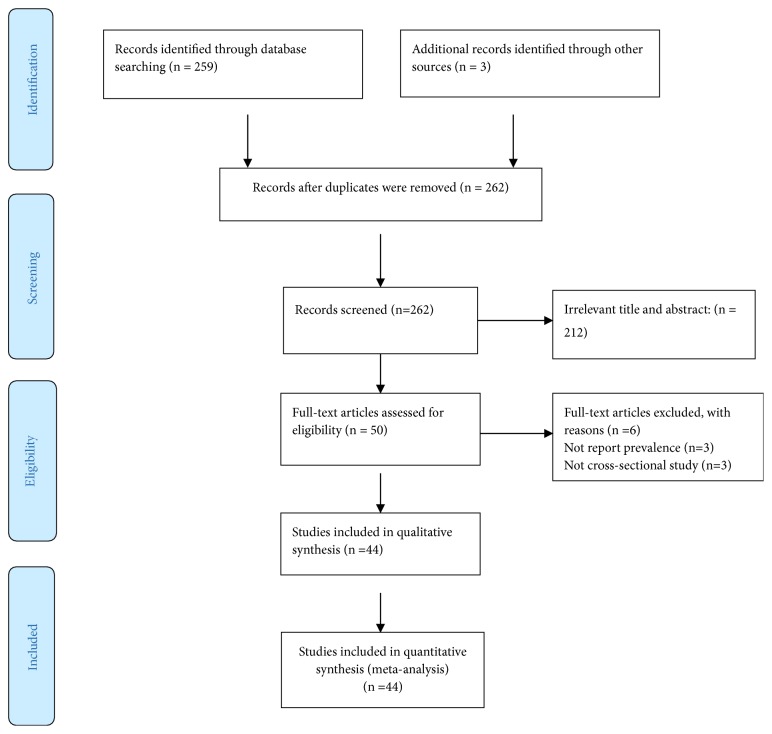
Flow diagram of studies reviewed, screened, and included.

A total of 262 citations were retrieved through electronic database search. Of these, 212 were excluded based on screening of titles and abstracts. From the remaining fifty articles, six articles were excluded (three articles are not cross-sectional studies and three have no prevalence data). Finally, 44 studies were found to be eligible and were included in the meta-analysis (Figure 1). Because of the high heterogeneity observed across studies (Q=3930.11, p <0.0001;* I*^*2*^= 98.9% and Tau-squared, p= 2.269), a random-effects model was used for the meta-analysis. Eggers regression intercept test indicated no evidence of publication bias (p =0.154) (Figure 3).

### 3.2. Study Characteristics

A total of 44 studies were considered eligible for quantitative syntheses (Table 1). Included articles were published from 1999 to 2018 across sub-Saharan Africa—East, West, Central, and Southern Africa. The total number of study subjects involved in the systematic review and meta-analysis was 175,546. From the total study population, regional study populations included in the review were 80 809 from Eastern region, 43 480 from West Africa region, 25 811 from Central Africa region, and 26 046 from Southern Africa. The study population among individual studies varied from 165 to 39 698 and were conducted between the years 1999 to 2018 (Table 1). All studies utilized blood specimens for diagnosis of syphilis (Table 1). All the included studies utilized data which were collected from health facilities. The reported prevalence of syphilis among pregnant women ranged from 0 to 22.1%. RPR, TPHA ICT, and ELISA were used for the diagnosis of syphilis infection (Table 1).

### 3.3. Meta-Analysis of Syphilis

**Figure 2:**
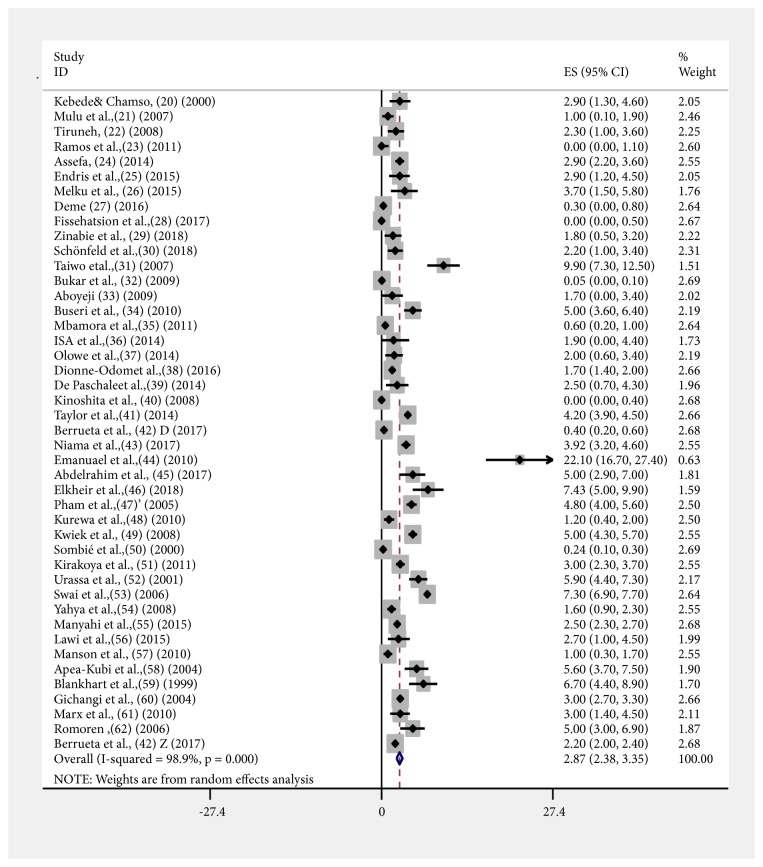
The meta-analysis and forest plot of prevalence of syphilis from 1999 to 2018.

**Figure 3:**
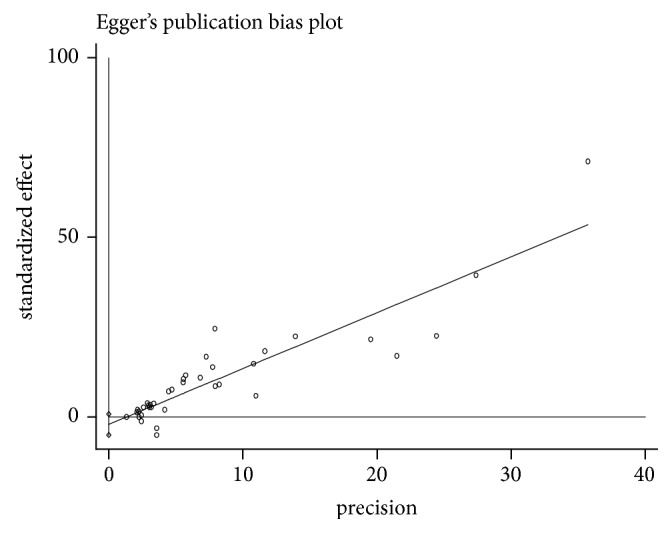
Funnel plot of standard error by logit event rate.

**Figure 4:**
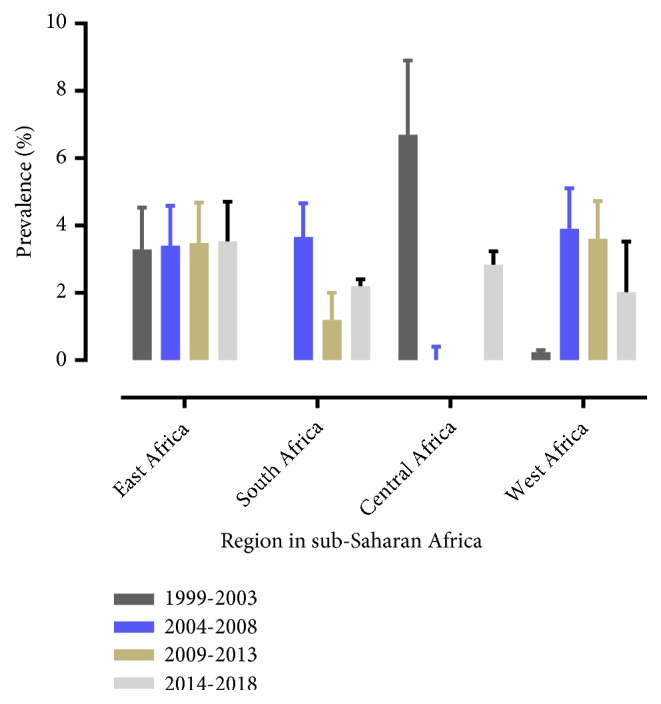
Pattern of the prevalence of syphilis in the four subregions in sub-Saharan Africa from 1999 to 2018.

The analysis of 44 studies, according to the Der Simonian-Laird random-effects model, revealed that the pooled prevalence of syphilis in sub-Saharan Africa was 2.87% (95% CI: 2.38-3.35) (Figure 2). Pooled prevalence of syphilis varied by region—2.17% (95% CI: 2.06-2.29) for Eastern Africa, 0.16% (95% CI: - 0.12-0.20) for Western Africa, 1.04% (95%CI: 0.52-1.17) for Central Africa, and 2.49% (95%CI: 2.31-2.68) for Southern Africa (Figure S1 and Table 2), while grouping the pooled prevalence was by every five years—0.29% (95% CI: 0.19-0.39) during 1999-2003, 2.12% (95% CI: 1.97-2.26) during 2004-2008, 0.09% (95%CI: 0.04-0.14) during 2009-2013, and 1.72% (95% CI: 1.63-1.81) during the 2014-2018 period (Figures S2 and S3 and Table 2). In addition, we performed a metaregression analysis to identify the possible sources of heterogeneity, by considering different factors associated with the heterogeneity, such as publication year geographic region and diagnostic tests, but none of these variables was found to be statistically significant. The prevalence of syphilis showed a decreasing trend in Southern, Central, and West African regions but showed a similar trend in the Eastern African region (Figure 4). The subgroup analysis was done on the prevalence of syphilis by type of diagnostic test: nontreponemal test (NTT): RPR&VDRL; treponemal test (TT): TPHA&EIA; and point of care test (POT): ICT. Pooled prevalence of syphilis was 3.77% (95%CI: 2.83–4.71) using NTT; 2.23% (95%CI: 1.77–2.68) using NTT and TT; 0.63% (95%CI: 0.18–1.43) using TT; 2.33% (95%CI: 0.93–3.72) using POT; and 3.91% (95%CI: 1.66–6.16) using POT&TT (Table 2 and Figure S3).

## 4. Discussion

Syphilis is one of the easily preventable and treatable sexually transmitted infections but continues to exert a high burden worldwide especially in sub-Saharan Africa. Prevention and control of syphilis is crucial for pregnant women. Antenatal care (ANC) follow-up screening plays a great role in diagnosing syphilis early. However, the actual burden of syphilis infection during pregnancy is not well known especially in resource poor settings where diagnosis is made based on clinical signs and symptoms, short of diagnostic laboratory tests. Clinical diagnosis of syphilis infection is subject to underestimating the real burden as clinically asymptomatic cases would be unaccounted for.

The current meta-analysis estimated the pooled prevalence of syphilis, 2.87% (95% CI: 2.38-3.35) in sub-Saharan Africa using 44 studies. Similar prevalence (2.7%) was reported in a previous systematic review conducted in sub-Saharan Africa among pregnant women [63]. However, studies reported a higher prevalence of syphilis infection in East and South African regions (4.5%) [64], among incarcerated women (6.10%) [65] and among aboriginal populations in Australia (16.8%) [66]. The differences in the prevalence of syphilis mainly reflect the risk profiles of the populations included in the studies. Moreover, the findings indicate that certain regions in sub-Saharan Africa exhibit a particularly higher prevalence of syphilis.

The current review also showed that the prevalence of syphilis infection during pregnancy has remained to be high in the East African region. Similar rise in the prevalence of syphilis has been reported from the developed world [7]. The persistently high prevalence of syphilis in East Africa could be related to affordability of cost for screening and the relatively weekly ANC follow up [67], unavailability of treatment or reinfection from an untreated sexual partner [68]. The high prevalence of syphilis in the region is a particular concern as syphilis infection during pregnancy frequently leads to unfavorable pregnancy outcomes including fetal/neonatal mortality and morbidity because of congenital syphilis.

Even though a decreasing trend was observed, the prevalence of syphilis infection during pregnancy was the highest in Southern Africa followed by East Africa. The prevalence in this region was fifteen times higher than that of West Africa, even though the number of identified studies in Southern Africa was small further limiting the scope of the review to understand the true prevalence in the region. Similarly, a high prevalence of syphilis infection during pregnancy was reported from East and South Africa [64]. The regional differences in the prevalence of syphilis infection in pregnant women could be because of differences in the strength of programs for primary prevention, quality of ANC follow-up programs and availability of diagnostic and therapeutic interventions in the health system.

Higher pooled prevalence of* syphilis* was reported by NTT tests. Pooled prevalence of syphilis during pregnancy by using studies which used TT tests for the diagnosis was low. This could be possibly explained by the high sensitivity and lower specificity of NTT test for the diagnosis of syphilis infection. The high pooled prevalence by NTT may be due to false-positive reactions related to either acute or chronic infection. False-positive reactions to NTT tests could be due to several acute and chronic disorders including acute febrile illnesses, vaccination, pregnancy itself, connective tissue disorders, cancer, Chagas disease, or tuberculosis [69, 70].

The current review and meta-analysis used data from urban populations who have better access to preventive and therapeutic programs and services for syphilis infection. This may underestimate the real burden of syphilis infection in the rural population of pregnant women. Moreover, most of studies which were included in the analysis were facility based studies, and the data might not be representative of population/community-based prevalence of syphilis among pregnant women. As diagnostic tests are usually applied for women who have symptoms, the current prevalence could be considered as an underestimation because of unavailability of studies targeting asymptomatic individuals to enable a more accurate estimation of prevalence of syphilis. Furthermore, as the included studies used different diagnostic tests which have varying sensitivity and specificity, heterogeneity of the results might have biased the true estimate of syphilis in the region. Additionally, the review protocol for the current review has not been registered online and could be considered as a limitation. As any disagreements between the reviewers were resolved through discussion, agreement between the reviewers was not assessed using the Cohens Kappa coefficient which might have limited objectivity.

In conclusion, the current review revealed that prevalence of syphilis among pregnant women is high in sub-Saharan Africa. The prevalence is particularly high in the Southern and East African regions. The review highlights that syphilis infection during pregnancy is still a high burden necessitating implementation of programs that can facilitate primary prevention and also intensify diagnosis and treatment of pregnant mothers diagnosed with syphilis.

## Figures and Tables

**Table 1:** General characteristics of the included studies in systematic review and meta-analysis from 1999 to 2018.

Author & ref.	Pub. year	Study area	Sample size	Number(%)	Specimen	DX. methods	Qua (9%)
Kebede & Chamso, [20]	2000	Ethiopia	410	12(2.9)	Blood	VDRL	7
Mulu et al., [21]	2007	Ethiopia	480	5(1.0)	Blood	RPR&TPHA	7
Tiruneh, [22]	2008	Ethiopia	480	11(2.3)	Blood	RPR &TPHA	7
Ramos et al., [23]	2011	Ethiopia	165	0(0.0)	Blood	ELISA	5
Assefa, [24]	2014	Ethiopia	2385	69(2.9)	Blood	RPR& TPHA	8
Endris et al., [25]	2015	Ethiopia	385	11(2.9)	Blood	RPR& TPHA	8
Melku et al., [26]	2015	Ethiopia	300	11(3.7)	Blood	RPR&TPHA	8
Deme [27]	2016	Ethiopia	574	2(0.3)	Blood	RPR	7
Fissehatsion et al., [28]	2017	Ethiopia	403	0(0.0)	Blood	RICT	8
Zinabie et al., [29]	2018	Ethiopia	385	7(1.8)	Blood	VDRL	8
Schönfeld et al., [30]	2018	Ethiopia	580	13(2.2)	blood	RICT	7
Taiwo etal., [31]	2007	Nigeria	505	50(9.9)	Blood	RPR&TPHA	6
Bukar et al., [32]	2009	Nigeria	18101	9(0.1)	Blood	RPR&TPHA	7
Aboyeji [33]	2009	Nigeria	230	4(1.7)	Blood	RPR	6
Buseri et al., [34]	2010	Nigeria	1000	50(5.0)	Blood	RICT &TPHA	5
Mbamora et al., [35]	2011	Nigeria	1393	8(0.6)	Blood	ELISA&TPHA	7
ISA et al., [36]	2014	Nigeria	108	2(1.9)	Blood	RICT	6
Olowe et al., [37]	2014	Nigeria	394	8(2.0)	Blood	RPR&TPHA	5
Dionne-Odomet al., [38]	2016	Cameron	7069	120(1.7)	Blood	RICT	7
De Paschaleet al., [39]	2014	Benin	283	7(2.5)	Blood	EIA	5
Kinoshita et al., [40]	2008	DRC	529	0(0.0)	Blood	RPR&TPHA	6
Taylor et al., [41]	2014	DRC	17669	742(4.2)	Blood	RICT &RPR	8
Berrueta et al., [42] D	2017	DRC	4153	17(0.4)	Blood	RICT	5
Niama et al., [43]	2017	DRC	2979	117(3.9)	Blood	RPR	7
Emanuael et al., [44]	2010	Sudan	231	51(22.1)	Blood	RPR	9
Abdelrahim et al., [45]	2017	Sudan	426	21(5.0)	Blood	RPR	6
Elkheir et al., [46]	2018	Sudan	444	33(7.4)	Blood	RICT	9
Pham et al., [47]	2005	Zimbabwe	2969	143(4.8)	Blood	RPR	8
Kurewa et al., [48]	2010	Zimbabwe	691	8(1.2)	Blood	RPR&TPHA	9
Kwiek et al., [49]	2008	Malawi	3824	191(5.0)	Blood	RPR&TPHA	8
Sombié et al., [50]	2000	Burkina Faso	10980	26(0.2)	Blood	RPR &TPHA	9
Kirakoya et al., [51]	2011	Burkina Faso	2136	64(3.0)	Blood	RPR	5
Urassa et al., [52]	2001	Tanzania	1058	62(5.9)	Blood	VDRL&TPHA	7
Swai et al., [53]	2006	Tanzania	17323	1265(7.3)	Blood	RPR	8
Yahya et al., [54]	2008	Tanzania	1296	21(1.6)	Blood	RPR&TPHA	9
Manyahi et al., [55]	2015	Tanzania	39698	992(2.5)	Blood	RPR	7
Lawi et al., [56]	2015	Tanzania	331	9(2.7)	Blood	RPR& RICT	8
Manson et al., [57]	2010	Guinea	711	7(1.0)	Blood	RPR	6
Apea-Kubi et al., [58]	2004	Ghana	570	32(5.6)	Blood	TPHA	6
Blankhart et al., [59]	1999	CAR	481	32(6.7)	Blood	RPR	8
Gichangi et al., [60]	2004	Kenya	12414	372(3.0)	Blood	RPR	6
Marx et al., [61]	2010	Kenya	441	13(3.0)	Blood	RPR&TPHA	9
Romoren, [62]	2006	Botswana	465	23(5.0)	Blood	RPR&TPHA	6
Berrueta et al., [42] Z	2017	Zambia	18097	398(2.2)	Blood	RPR	5

RPR=rapid plasma regain, TPHA=treponema palladium hama agglutination inhibition test, VDRL=venereal disease research laboratory, RICT= rapid immunochromatography test, Pub-year=publication year, Ref=reference, CAR=Central African Republic, DRC=Democratic Republic of Congo.

**Table 2:** Subgroup meta-analysis of syphilis prevalence in sub-Saharan Africa from 1999 to 2018.

Categories	Subgroup	No. of studies included	Pre. (%) (95% CI	* I* ^2^
Region	East Africa	21	3.25(2.24-4.25)	98.4
	West Africa	13	1.86(1.39-2.32)	96.5
	Central Africa	5	2.85(1.02-4.68)	99.4
	Southern Africa	5	3.35(2.04-5.09)	96.2

Year of study	1999-2003	4	3.86(0.36-7.36)	97.0
	2004-2008	11	4.05(2.18-5.91)	99.2
	2009-2013	10	2.07(1.18-2.97)	95.9
	2014-2018	19	2.47(2.38-3.35)	97.8

*Serologic tests*	NTT	16	3.77 (2.83-4.71)	98.2
	NTT & TT	16	2.23 ( 1.77-2.68)	96.9
	TT	3	0.63(0.18-1.43)	75.3
	POT	7	2.33 (0.93-3.72)	99.0
	POT & TT	2	3.91(1.66-6.16)	98.9

*NB*: TT=treponemal test, NTT= nontreponemal test, POT= point of care test, CI = confidence interval.

## Data Availability

No additional data are available.

## Supplementary Materials

Supplementary Materials Table S1 presents PRISMA Group (2009) Preferred Reporting Items for Systematic Reviews and Meta-Analyses. Table S2 shows the study design and quality assessment of the studies included in systematic review and meta-analysis of syphilis in sub-Saharan Africa. Figure S1 represents the subgroup meta-analysis by geographical region. The forest plot presentation indicates the prevalence of syphilis from 1999 to 2018 in each region, whereas Figure S2 shows the subgroup meta-analysis by year of study. The forest plot indicates the prevalence of syphilis during each 5-year period. Figure S3 presents the subgroup meta-analysis by laboratory diagnostic test. The forest plot shows the prevalence of syphilis by each diagnostic test from 1999 to 2018. Metareg S1 presents the metaregression outputs for the estimates. Syntax S2 presents the syntax used for the search of databases.
