# Supplementary material for: Prevalence of Syphilis among Pregnant Women in Sub-Saharan Africa: A Systematic Review and Meta-Analysis
Source: Biomed Res Int. 2019 Jul 16;2019:4562385. doi: 10.1155/2019/4562385 (PMC6662498; doi:10.1155/2019/4562385)
Supplement: Supplementary Materials — Table S1 presents PRISMA Group (2009) Preferred Reporting Items for Systematic Reviews and Meta-Analyses. Table S2 shows the study design and quality assessment of the studies included in systematic review and meta-analysis of syphilis in sub-Saharan Africa. Figure S1 represents the subgroup meta-analysis by geographical region. The forest plot presentation indicates the prevalence of syphilis from 1999 to 2018 in each region, whereas Figure S2 shows the subgroup meta-analysis by year of study. The forest plot indicates the prevalence of syphilis during each 5-year period. Figure S3 presents the subgroup meta-analysis by laboratory diagnostic test. The forest plot shows the prevalence of syphilis by each diagnostic test from 1999 to 2018. Metareg S1 presents the metaregression outputs for the estimates. Syntax S2 presents the syntax used for the search of databases. [file 4562385.f1.zip › 4562385.f1/Tadesse_Table S2_Quality Assessment.docx]

Table S2: Study design and quality assessment of the studies included in systematic review and meta-analysis of syphilis in sub Saharan Africa

| Author(s) ref | Q1 | Q2 | Q3 | Q4 | Q5 | Q6 | Q7 | Q8 | Q9 | Quality score (9%) |
| --- | --- | --- | --- | --- | --- | --- | --- | --- | --- | --- |
| Kebede& Chamso, (20) | Y | Y | Y | Y | Y | Y | Y | NA | NR | 7 |
| Mulu et al.,(21) | Y | Y | Y | Y | Y | Y | NR | NA | NR | 7 |
| Tiruneh, (22) | Y | Y | Y | Y | Y | Y | ? | Y | ? | 7 |
| Ramos et al.,(23) | Y | Y | NR | Y | Y | Y | NR | NA | NR | 5 |
| Assefa, (24) | Y | NR | Y | Y | Y | Y | Y | Y | Y | 8 |
| Endris et al.,(25) | Y | Y | Y | Y | Y | Y | NR | Y | Y | 8 |
| Melku et al., (26) | Y | Y | Y | Y | Y | Y | NR | Y | Y | 8 |
| Deme (27) | Y | Y | Y | Y | Y | Y | NR | Y | NR | 7 |
| Fissehatsion et al.,(28) | Y | Y | Y | Y | Y | Y | Y | Y | NR | 8 |
| Zinabie et al., (29) | Y | Y | Y | Y | Y | Y | Y | Y | NR | 8 |
| Schönfeld et al.,(30) | Y | Y | Y | Y | Y | Y | NR | Y | NR | 7 |
| Taiwo etal.,(31) | Y | Y | Y | Y | Y | Y | NR | NA | NR | 6 |
| Bukar et al., (32) | Y | Y | Y | Y | Y | Y | NR | Y | NR | 7 |
| Aboyeji (33) | Y | Y | Y | Y | Y | Y | NR | NA | NR | 6 |
| Buseri et al., (34) | Y | Y | Y | NR | Y | Y | NR | NA | NR | 5 |
| Mbamora et al.,(35) | Y | Y | Y | Y | Y | Y | NR | NA | Y | 7 |
| ISA et al.,(36) | Y | Y | Y | Y | Y | Y | NR | NA | NR | 6 |
| Olowe et al.,(37) | Y | Y | Y | Y | NR | Y | NR | NA | NR | 5 |
| Dionne-Odomet al.,(38) | Y | Y | Y | Y | Y | Y | NR | Y | NR | 7 |
| De Paschaleet al.,(39) | Y | NR | Y | Y | Y | Y | NR | NA | NR | 5 |
| Kinoshita et al., (40) | Y | Y | Y | Y | Y | Y | NR | NA | NR | 6 |
| Taylor et al.,(41) | Y | Y | Y | Y | Y | Y | Y | Y | NR | 8 |
| Berrueta et al., (42) D | Y | Y | NR | Y | Y | Y | NR | NA | NR | 5 |
| Niama et al.,(43) | Y | Y | Y | Y | Y | Y | NR | NA | Y | 7 |
| Emanuael et al.,(44) | Y | Y | Y | Y | Y | Y | Y | Y | Y | 9 |
| Abdelrahim et al., (45) | Y | Y | Y | Y | Y | Y | NR | NA | NR | 6 |
| Elkheir et al.,(46) | Y | Y | Y | Y | Y | Y | Y | Y | Y | 9 |
| Pham et al.,(47)’ | Y | Y | Y | Y | Y | Y | Y | Y | NR | 8 |
| Kurewa et al.,(48) | Y | Y | Y | Y | Y | Y | Y | Y | Y | 9 |
| Kwiek et al., (49) | Y | Y | Y | Y | Y | Y | NR | Y | Y | 8 |
| Sombié et al.,(50) | Y | Y | Y | Y | Y | Y | Y | Y | Y | 9 |
| Kirakoya et al., (51) | Y | Y | NR | Y | Y | Y | NR | NA | NR | 5 |
| Urassa et al., (52) | Y | Y | Y | Y | Y | Y | NR | Y | NR | 7 |
| Swai et al.,(53) | Y | Y | Y | Y | Y | Y | Y | Y | NR | 8 |
| Yahya et al.,(54) | Y | Y | Y | Y | Y | Y | Y | Y | Y | 9 |
| Manyahi et al.,(55) | Y | Y | Y | Y | Y | Y | NR | Y | NR | 7 |
| Lawi et al.,(56) | Y | Y | Y | Y | Y | Y | NR | Y | Y | 8 |
| Manson et al., (57) | Y | Y | Y | Y | Y | Y | NR | NA | NR | 6 |
| Apea-Kubi et al.,(58) | Y | NR | Y | Y | Y | Y | Y | NA | NR | 6 |
| Blankhart et al.,(59) | Y | Y | Y | Y | Y | Y | Y | NA | Y | 8 |
| Gichangi et al., (60) | Y | Y | Y | NR | Y | Y | NR | NA | Y | 6 |
| Marx et al., (61) | Y | Y | Y | Y | Y | Y | Y | Y | Y | 9 |
| Romoren ,(62) | Y | Y | Y | Y | Y | Y | NR | NA | NR | 6 |
| Berrueta et al., (42) Z | Y | Y | NR | Y | Y | Y | NR | NA | NR | 5 |

**Key:** **Y**= Yes; **NR**= Not reported, **NA**=Not appropriate

**Question codes:**

1. Was the sample frame appropriate to address the target population?

2. Were study participants sampled in an appropriate way?

3. Was the sample size adequate?

4. Were the study subjects and the setting described in detail?

5. Wasthedataanalysisconductedwithsufficientcoverageoftheidentified sample?

6. Were valid methods used for the identification of the condition?

7. Was the condition measured in a standard, reliable way for all participants?

8. Was there appropriate statistical analysis?

9. Was the response rate adequate, and if not, was the low response rate managed appropriately?
