# Supplementary figures and images for: Prevalence of Syphilis among Pregnant Women in Sub-Saharan Africa: A Systematic Review and Meta-Analysis
Source: Biomed Res Int. 2019 Jul 16;2019:4562385. doi: 10.1155/2019/4562385 (PMC6662498; doi:10.1155/2019/4562385)

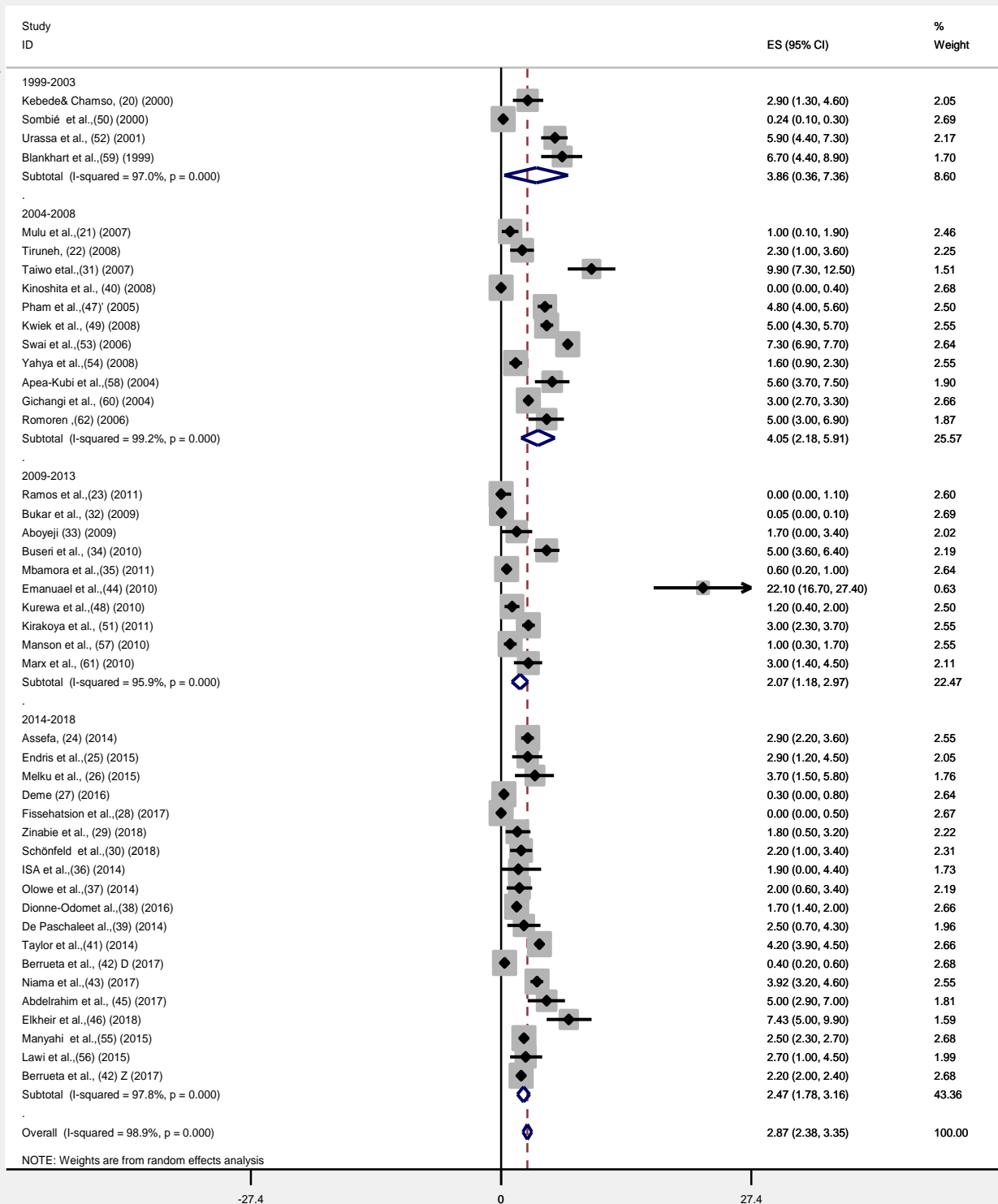

Supplement: Supplementary Materials — Table S1 presents PRISMA Group (2009) Preferred Reporting Items for Systematic Reviews and Meta-Analyses. Table S2 shows the study design and quality assessment of the studies included in systematic review and meta-analysis of syphilis in sub-Saharan Africa. Figure S1 represents the subgroup meta-analysis by geographical region. The forest plot presentation indicates the prevalence of syphilis from 1999 to 2018 in each region, whereas Figure S2 shows the subgroup meta-analysis by year of study. The forest plot indicates the prevalence of syphilis during each 5-year period. Figure S3 presents the subgroup meta-analysis by laboratory diagnostic test. The forest plot shows the prevalence of syphilis by each diagnostic test from 1999 to 2018. Metareg S1 presents the metaregression outputs for the estimates. Syntax S2 presents the syntax used for the search of databases. [file 4562385.f1.zip › 4562385.f1/Tadesse_Fig S2.pdf]
